# Heart Rate Variability and Intrinsic Autonomic Coupling in Ehlers–Danlos Syndrome

**DOI:** 10.7759/cureus.98693

**Published:** 2025-12-08

**Authors:** Waqas Alauddin, Prajakta M Radke, Nithya Janardhana, Ishita Singh, Ayush Sharma, Shashwat Arora, Brishabh R Prajesh, Rishika Shree, Zaki Shaikh

**Affiliations:** 1 Physiology, Naraina Medical College and Research Centre, Kanpur, IND; 2 Physiology, Mahatma Gandhi Mission Medical College, Nerul, IND; 3 Medicine, Adichunchanagiri Institute of Medical Sciences, Karnataka, IND; 4 Medicine, Lala Lajpat Rai Memorial Medical College, Meerut, IND; 5 Medicine, Amar Shaheed Jodha Singh Atayiya Thakur Dariyao Singh Medical College, Fatehpur, IND; 6 Medicine, Naraina Medical College and Research Centre, Kanpur, IND

**Keywords:** autonomic nervous system, cardiovascular autonomic dysfunction, dysautonomia, ehlers-danlos syndrome, heart rate variability, orthostatic intolerance, parasympathetic nervous system, parasympathetic tone, postural tachycardia syndrome, sympathetic nervous system

## Abstract

Background

Ehlers-Danlos syndrome (EDS) encompasses a group of connective tissue disorders often extending beyond musculoskeletal involvement. Emerging evidence indicates a high prevalence of cardiovascular autonomic dysfunction in this population, yet systematic physiologic evaluations remain limited.

Objective

To characterize cardiovascular autonomic function in EDS using standardized autonomic testing and heart rate variability (HRV) indices, and to explore intrinsic autonomic coupling by correlating resting heart rate with HRV parameters.

Methods

This cross-sectional study included 30 clinically diagnosed patients with EDS and 30 age- and sex-matched healthy controls. Short-term HRV analysis (five-minute supine ECG) and standard autonomic testing, including head-up tilt, were performed under controlled laboratory conditions. HRV indices were derived using Fast Fourier Transform (FFT) algorithms. Group differences were evaluated with independent t-tests, and correlations between resting heart rate and HRV measures were analyzed using Pearson’s correlation.

Results

Compared with controls, patients with EDS exhibited higher resting heart rate (87.3±11.6 vs 75.2±9.8 bpm), lower parasympathetic time-domain indices (standard deviation of normal-to-normal intervals or SDNN 35.4±9.7 vs 49.1±11.4 ms; root mean square of successive differences (RMSSD; 20.7±6.9 vs 31.6±8.8 ms), and altered frequency-domain markers (low frequency (LF) power 671±205 vs 542±176 ms²; high frequency (HF) power 174±81 vs 272±106 ms²; LF/HF ratio 3.7±1.3 vs 1.8±0.7). Orthostatic intolerance was observed in 16 (53.3%) of the patients with EDS versus three (10%) of the controls. Correlation analysis revealed that in EDS, resting HR correlated negatively with SDNN (r=-0.45, p=0.01), RMSSD (r=-0.52, p<0.01), and HF power (r=-0.39, p=0.03), while showing a positive correlation with LF/HF ratio (r=0.58, p<0.001).

Conclusion

Patients with EDS had autonomic dysregulation, with sympathetic predominance and diminished vagal modulation. The intrinsic coupling between resting heart rate and HRV indices suggests impaired cardiovascular autonomic integration. HRV profiling is a valuable noninvasive biomarker for early identification and longitudinal monitoring of autonomic dysfunction in EDS, potentially enhancing disease characterization and guiding individualized therapeutic strategies.

## Introduction

Ehlers-Danlos syndrome (EDS) refers to a heterogeneous group of inherited connective tissue disorders, typically characterized by skin hyperextensibility, joint laxity, and tissue fragility [1]. Estimates of prevalence vary widely, ranging from one in 5,000 to one in 20,000 individuals; however, hypermobile EDS likely represents the most common and underdiagnosed form [2]. While traditionally viewed as a musculoskeletal condition, it is now evident that EDS exerts systemic effects involving cardiovascular, neurological, gastrointestinal, and autonomic domains [3,4].

Among these multisystem features, autonomic dysfunction is increasingly recognized yet frequently overlooked in routine care [3,4]. Patients often experience symptoms such as palpitations, lightheadedness, presyncope, exercise intolerance, and persistent fatigue, with conditions like orthostatic intolerance and postural tachycardia syndrome (POTS) accounting for a considerable portion of this symptomatology [5,6]. Such disturbances substantially impair quality of life and may complicate disease management.

Heart rate variability (HRV) offers a noninvasive means of quantifying autonomic balance through the analysis of beat-to-beat fluctuations in cardiac rhythm [7,8]. Prior research suggests that individuals with EDS may exhibit reduced vagal modulation, increased sympathetic tone, and altered baroreflex sensitivity, although controlled studies remain sparse [9,10]. Complementary bedside tests, including deep breathing, the Valsalva maneuver, and tilt-table assessments, further illuminate autonomic responses under physiologic challenge [4-6].

In this context, the current study sought to investigate intrinsic autonomic coupling by investigating the relationship between resting heart rate and HRV parameters, as well as to thoroughly assess cardiovascular autonomic functioning in patients with EDS with standardized autonomic testing as well as HRV indices.

## Materials and methods

This cross-sectional study was carried out at the Naraina Medical College and Hospital, Kanpur, India, between January 2024 and January 2025. Ethical approval was obtained from the Institutional Ethics Committee of Naraina Medical College and Research Center (approval No. NMCRC/IEC/2023/012). Thirty patients with EDS who satisfied the 2017 International Classification criteria [1] were gathered from the medicine department. An equal number of healthy volunteers, who were matched for age and sex, served as controls. Participants, whose ages ranged from 18 to 45, were eligible if they provided written informed consent. Conditions that were excluded included diabetes mellitus, hypertension, thyroid dysfunction, structural heart disease, and current use of drugs that have autonomic effects, such as fludrocortisone, beta-blockers, and selective serotonin reuptake inhibitors.

Autonomic function testing (AFT) protocol

Participants reported to the AFT laboratory of the Department of Physiology at approximately 9:00 AM after consuming a light breakfast, without tea or coffee intake. Following informed consent, demographic and anthropometric data, including age, height, body weight, body mass index (BMI), and waist-to-hip ratio (WHR), were recorded. The laboratory environment was maintained at a constant temperature of 25°C throughout the recordings. Blood pressure was measured according to standard protocols in the AFT laboratory. After 15 minutes of supine rest on a couch, a five-minute electrocardiogram (ECG) was obtained for short-term HRV analysis. Lead II ECG was recorded at a sampling rate of 1000 samples/second using a BIOPAC MP100 data acquisition system (Biopac Systems Inc., Goleta, CA, US). 

HRV was analyzed using HRV Analysis Software (AcqKnowledge Software, Biopac Systems Inc., Goleta, CA, US) with the Fast Fourier Transform (FFT) algorithm. Time-domain indices included mean RR interval, root mean square of successive differences (RMSSD), and the percentage of such intervals (pNN50). Frequency-domain parameters assessed were total power (TP) and low frequency/high frequency (LF/HF) ratio [9,11].

Head-up tilt test

Participants were tilted to 70° for 10 minutes. Orthostatic intolerance was diagnosed if heart rate rose ≥30 bpm (≥40 bpm in adolescents) within 10 minutes of tilt, in the absence of hypotension [5,10].

Statistical analysis

The continuous variables' standard deviations (SD) were shown after their mean values. Independent sample t-tests were used to assess if the measured parameters varied significantly across groups. Within the EDS group, Pearson’s correlation coefficients were calculated to assess the relationship between resting heart rate and HRV indices. A p-value of less than 0.05 was considered statistically significant. IBM SPSS Statistics for Windows, Version 25 (Released 2019; IBM Corp., Armonk, New York, United States) was used for all analyses.

## Results

Autonomic indices and baseline features

Table 1 displays the demographic and baseline autonomic parameters of patients with EDS and the controls.

**Table 1:** Baseline characteristics and autonomic indices in patients with EDS and controls The data is displayed as a percentage or mean ± standard deviation (SD). LF: low frequency; HF: high frequency; HRV: heart rate variability; HR: heart rate; SDNN: standard deviation of normal to normal intervals; RMSSD: root mean square of successive differences. *p-value less than 0.05 is significant.

Parameter	Patients with EDS (n=30) (mean ± SD)	Controls (n=30) (mean ± SD)	p-value
Age (years)	29.8 ± 8.7	28.9 ± 9.1	0.72
Female (%)	66%	63%	0.81
Resting HR (bpm)	87.3 ± 11.6	75.2 ± 9.8	<0.001*
SDNN (ms)	35.4 ± 9.7	49.1 ± 11.4	<0.01*
RMSSD (ms)	20.7 ± 6.9	31.6 ± 8.8	<0.01*
LF power (ms²)	671 ± 205	542 ± 176	0.03*
HF power (ms²)	174 ± 81	272 ± 106	0.02*
LF/HF ratio	3.7 ± 1.3	1.8 ± 0.7	<0.001*
Orthostatic intolerance n (%)	16 (53.3%)	3 (10%)	<0.001*

The distribution of sexes and ages was similar across groups. The patients with EDS had a higher baseline heart rate (87.3±11.6 vs. 75.2±9.8 bpm) and lower parasympathetic time-domain markers (standard deviation of normal-to-normal intervals (SDNN): 35.4±9.7 vs. 49.1±11.4 ms; RMSSD: 20.7±6.9 vs. 31.6±8.8 ms; Table 1). Frequency-domain analysis revealed a higher LF/HF ratio (3.7±1.3 vs. 1.8±0.7), lower HF power (174±81 vs. 272±106 ms²), and higher LF power (671±205 vs. 542±176 ms²). Orthostatic intolerance was observed in 16 (53.3%) of patients with EDS and three (10%) of controls.

Correlation between resting heart rate and HRV parameters

The correlation analysis revealed an intrinsic autonomic coupling between resting heart rate and HRV parameters in patients with EDS. As shown in Table 2, resting HR correlated negatively with SDNN (r=-0.45, p=0.01), RMSSD (r=-0.52, p<0.01), and HF power (r=-0.39, p=0.03), reflecting reduced parasympathetic modulation at higher cardiac rates.

**Table 2:** Correlation between the resting heart rate and HRV parameters in patients with EDS LF: low frequency; HF: high frequency; HRV: heart rate variability; HR: heart rate; SDNN: standard deviation of normal to normal intervals; RMSSD: root mean square of successive differences. Correlation analysis demonstrates a strong inverse association between resting heart rate and vagally mediated HRV indices (SDNN, RMSSD, HF power) and a positive association with LF/HF ratio, suggesting sympathetic dominance and impaired vagal modulation in EDS. Negative correlations denote reduced parasympathetic activity with increasing heart rate. *p<0.05 considered significant.

HRV parameter	Pearson’s r	p-value
SDNN (ms)	–0.45	0.01*
RMSSD (ms)	–0.52	<0.01*
LF power (ms²)	0.29	0.12
HF power (ms²)	–0.39	0.03*
LF/HF ratio	0.58	<0.001*

Conversely, the resting heart rate exhibited a strong positive correlation with LF/HF ratio (r=0.58, p<0.001) and moderate correlation with LF power (r=0.29, p=0.12), signifying a shift toward sympathetic predominance.

## Discussion

This study provides clear evidence of intrinsic autonomic coupling abnormalities in EDS. Compared with controls, patients with EDS showed higher resting heart rates, reduced parasympathetic markers, and elevated LF/HF ratios indicative of sympathetic predominance. Importantly, the resting heart rate exhibited a strong negative correlation with SDNN, RMSSD, and HF power, reflecting diminished vagal modulation at higher heart rates.

Several mechanisms likely contribute to dysautonomia in EDS. Laxity of venous connective tissue promotes excessive pooling, reducing venous return and triggering compensatory sympathetic activation [5,12]. Impaired baroreceptor function, altered vascular compliance, and small fiber neuropathy have also been implicated [10,13]. Collectively, these pathways explain the sympathetic predominance and parasympathetic withdrawal observed in our study. Such coupling between cardiac chronotropy and HRV indicates a fixed sympathetic drive even at rest. This may arise from altered baroreflex sensitivity, vascular laxity, and impaired venous return secondary to connective tissue fragility, all of which favor chronic sympathetic activation. The positive association between resting heart rate and the LF/HF ratio reinforces this notion.

Our findings are consistent with earlier reports highlighting autonomic dysfunction in EDS. Gazit et al. [13] described increased orthostatic intolerance, while De Wandele et al. [8,14] demonstrated reduced vagal modulation in hypermobile EDS. Hakim et al. [5] and Alauddin et al. [10] further established that patients with EDS have a strong association with postural orthostatic tachycardia syndrome (POTS), emphasizing autonomic nervous system dysfunction. Our results are aligned with previous studies, which also reported parasympathetic withdrawal and elevated sympathetic tone in hypermobile EDS [5,13,14]. The correlation approach applied here builds on previous research by demonstrating that intrinsic autonomic coupling is a physiologic interaction pattern between resting cardiac rate and autonomic balance, and can act as a potential noninvasive biomarker of dysautonomia severity in EDS.

The clinical relevance of this study is that recognizing autonomic impairment in EDS has important implications for patient care. Dysautonomia contributes significantly to morbidity and reduction in quality of life [5,10]. Early identification can prompt tailored interventions such as increased fluid and salt intake, compression therapy, and structured exercise regimens [6]. Selected patients may benefit from pharmacological approaches, including beta-blockers, ivabradine, midodrine, or fludrocortisone [6]. A coordinated, multidisciplinary approach is recommended to optimize outcomes.

The use of well-matched controls and a standardized HRV analysis, which reduced potential confounding, are two of this study's strengths. Nevertheless, the cross-sectional design, lack of genetic subtyping, and comparatively small sample size restrict causal inference. To assess targeted therapeutic approaches and clarify underlying mechanisms, future longitudinal studies with larger cohorts are necessary.

## Conclusions

Patients with EDS reveal a distinct pattern of autonomic dysregulation, characterized by sympathetic predominance and diminished vagal modulation. The observed intrinsic coupling between resting heart rate and HRV indices underscores impaired cardiovascular autonomic integration, reflecting a state of physiologic instability. These findings highlight HRV profiling as a valuable, noninvasive biomarker for the early identification and longitudinal monitoring of autonomic dysfunction in EDS. Incorporating autonomic assessment into clinical evaluation may enhance disease characterization and guide individualized therapeutic strategies.
